# Supplementary material for: Assessing Misophonia in Young Adults: The Prevalence and Psychometric Validation of the MisoQuest Questionnaire
Source: Healthcare (Basel). 2024 Sep 20;12(18):1888. doi: 10.3390/healthcare12181888 (PMC11430847; doi:10.3390/healthcare12181888)
Supplement: Supplementary file 1 [file healthcare-12-01888-s001.zip › File S2. QUESTIONAIRE_1_MISOQUEST_healthcare.pdf]

MISOQUEST – QUESTIONÁRIO PARA AVALIAÇÃO DE INTOLERÂNCIA A SONS

AUTORES: *Siepsiak, M., Śliwerski, A., Dragan, W. Ł*
TRADUZIDO E ADAPTADO POR: Autores

Algumas pessoas são mais sensíveis a certos sons do que outras. Existem sons, produzidos por pessoas ou objetos, que você os considera muito irritantes? Indique a seguir quanto você concorda ou discorda com as afirmativas seguintes, usando a escala abaixo:

1. Eu discordo totalmente.

2. Eu discordo.

3. Não sei dizer

4. Eu concordo

5. Eu concordo totalmente.

1	Alguns sons me irritam tanto que tenho dificuldade de controlar minhas emoções.	1	2	3	4	5
2	Sons desagradáveis me fazem sentir oprimido.	1	2	3	4	5
3	Eu fico ansioso(a) só de pensar em certos sons desagradáveis.	1	2	3	4	5
4	Eu acredito que minhas reações a certos sons são exageradas, mas eu não consigo me livrar delas.	1	2	3	4	5
5	Quando ouço sons desagradáveis, eu começo a sentir as emoções no meu corpo, tais como: transpiração, tensão muscular, dor e pressão no peito.	1	2	3	4	5
6	Eu começo a sentir raiva no momento em que vejo um objeto/animal/ pessoa que possa produzir algum som irritante naquele instante.	1	2	3	4	5
7	Eu me esforço muito em controlar as emoções quando ouço um som irritante.	1	2	3	4	5
8	Se eu puder, eu evito me encontrar com certas pessoas por causa dos sons que elas fazem.	1	2	3	4	5
9	Eu acho alguns sons produzidos por humanos insuportáveis.	1	2	3	4	5
10	Eu sinto que meu estado mental piora muito se eu não puder sair do lugar onde existe um som irritante.	1	2	3	4	5
11	Frequentemente, penso como eu poderia abafar sons irritantes.	1	2	3	4	5
12	Alguns sons irritantes produzem raiva instantânea em mim.	1	2	3	4	5
13	Eu me preocupo o quanto sons irritantes podem impactar minha vida futura.	1	2	3	4	5
14	Quando me encontro com outras pessoas, eu fico irritado(a) algumas vezes porque existem sons irritantes no ambiente.	1	2	3	4	5

MisoQuest – a questionnaire for assessing decreased sound tolerance

authors: Siepsiak, M., Śliwerski, A., Dragan, W. Ł

Some people are less sensitive to certain sounds, while other people are more sensitive to certain sounds. Are there any sounds which you find particularly burdensome? Please indicate how much you agree or disagree with the following statements using the following scale:

1 – I definitely do not agree
2 – I do not agree
3 – Hard to say
4 – I agree
5 – I definitely agree

1	Some sounds bother me so much that I have difficulty controlling my emotions.	1	2	3	4	5
2	Unpleasant sounds make me feel overwhelmed.	1	2	3	4	5
3	I become anxious at the mere thought of an unpleasant sound.	1	2	3	4	5
4	I believe that my reactions to sounds are exaggerated, but I can't get rid of them.	1	2	3	4	5
5	When I hear unpleasant sounds, I start sensing emotions in my body (e.g. I sweat, feel pain, feel pressure, my muscles tens).	1	2	3	4	5
6	I start feeling anger the moment I see a thing/animal/person that might make an unpleasant sound at any time.	1	2	3	4	5
7	I put a lot of effort into controlling emotions when I hear an unpleasant sound.	1	2	3	4	5
8	If I can, I avoid meeting with certain people because of the sounds they make.	1	2	3	4	5
9	I find some sounds made by the human body unbearable.	1	2	3	4	5
10	I feel that my mental state worsens if I cannot leave a place where there's an unpleasant sound.	1	2	3	4	5
11	I often think about how to drown out unpleasant sounds.	1	2	3	4	5
12	Some unpleasant sounds make me instantly angry.	1	2	3	4	5
13	I am scared that unpleasant sounds may impact my future.	1	2	3	4	5
14	When meeting with other people, I am sometimes irritated because of unpleasant sounds that are present.	1	2	3	4	5
